# Efficiency and Mechanism Evaluation of *Magnolia officinalis* Water Extract in Preventing Gastric Ulcer

**DOI:** 10.1155/2023/7901734

**Published:** 2023-04-06

**Authors:** Kuo-Ping Shen, Ching-Dong Chang, Meng-Hsun Hsieh, Hso-Chi Chaung

**Affiliations:** Department of Veterinary Medicine, Research Center for Animal Biologics, National Pingtung University of Science and Technology, Neipu, Pingtung 912, Taiwan

## Abstract

In this study we aimed at demonstrating the ability of *Magnolia officinalis* water extract to ameliorate gastric ulcers in *in vitro* and *in vivo* experiments. The gastric mucosa epithelial cell line, RGM 1, was pretreated with *Magnolia officinalis* water extract (0, 0.1, 1, 2, 5, or 10 mg/ml) and cultured in DMEM/F12 medium (pH 7.4) for 2 h and then in DMEM/F12 medium (pH 4.0) for 10 min. *Magnolia officinalis* water extract protected the cell viability and decreased reactive oxygen species formation by the acidic medium. In the *in vivo* experiment, *Magnolia officinalis* water extract (100 mg/kg) was administrated daily for 28 days in ICR mice via oral gavage, and then Shay's ulcer surgical method was performed to induce gastric ulcers. We analyzed the pH value of stomach acid and the pathological section, inflammation, and cannabinoid receptor type 2 (CB2) cDNA levels of the stomach. *Magnolia officinalis* water extract not only enhanced the pH value of stomach acid but also ameliorated the ulcer index and inflammation and increased CB2 expression effectively. These results suggest that *Magnolia officinalis* water extract might be used to decrease the incidence of gastric ulcer.

## 1. Introduction

Peptic ulcer disease (PUD) is attributable to multiple etiologies, including genetic characteristics, stress, lifestyle habits, smoking, drug use, and *Helicobacter pylori (H. pylori)* infection, which have been identified as major risk factors [1, 2]. PUD, consisting of duodenal ulcers and gastric ulcers, is a common disease in the outpatient department of gastroenterology and widely distributed across various ethnic and age groups. The prevalence of PUD is increased significantly in adults who smoke and have a history of long-term medication use [3]. In Taiwan, the prevalence rate of PUD is approximately 10% among people of any age. Moreover, 67% of patients with PUD have no particularly unremarkable symptoms [4]. Finally, proton pump inhibitors (PPIs) and anti *H. pylori* treatment do not significantly decrease the incidence of PUD [5].

Antacid agents, mucosal protectants (such as sucralfate), H2-antagonists (such as ranitidine), PPIs, and antibiotics of *H. pylori* are the most common drugs to treat gastric ulcers in modern medicine. However, in Taiwan, people use traditional herbal medicine to treat gastric disorders, and the efficacy of these treatments has been confirmed. For example, *Magnolia officinalis*, also called houpu, is a traditional herbal medicine in Asia for treating gastrointestinal disorders, such as indigestion, flatulence, duodenal ulcers, and gastric ulcers. Several reports have demonstrated that *Magnolia officinalis* ethanol or hexane extract possess several phenolic compounds (such as magnolol, honokiol) that can reduce the production of acidic gastric juice and inhibit the proton pump of gastric parietal cells. They also have antioxidation and anti-inflammation effects in an ethanol-induced gastric ulcer animal model [6, 7].

According to previous studies [6, 7], *Magnolia officinalis* extracts were most commonly made with ethanol or hexane. In this study, we investigated the protective effects of a unique and patented *Magnolia officinalis* water extract on gastric mucosa epithelial cells in acidic medium and gastric ulcers induced by Shay's ulcer surgical method in ICR mice.

## 2. Methods and Materials

### 2.1. *Magnolia officinalis* Extraction

**Figure 1:**
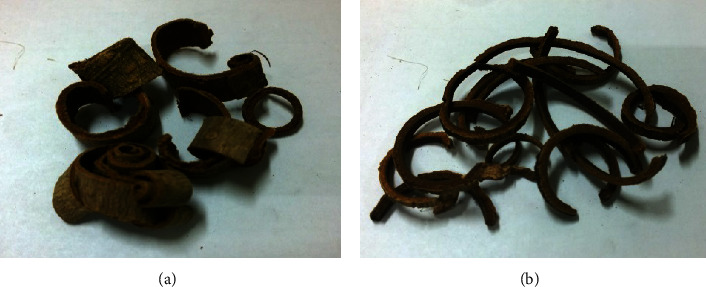
An introduction to *Magnolia officinalis* (houpu). *Magnolia officinalis*, also called houpu, is a traditional herbal medicine in Asia for treating gastrointestinal disorders, like indigestion, flatulence, duodenal ulcer, and gastric ulcer. Firstly, to collect the tree bark and root bark from *Magnolia officinalis* and to dry it (a), after soaking with ginger juice for a period of time, that is a complete medicinal material for clinical use (b). In this study, the houpu we used was soaked with ginger juice for a period of time.

We soaked 100 g of Cortex *Magnolia officinalis* (Figure 1) in 1000 ml of distilled pure water and boiled it for one hour. After filtering, the solution was evaporated at 60°C water bath *in vacuo* to remove the solvent (BUCHI Rotavapor R-200, Switzerland) and then lyophilized (EYELA, Freeze Dryer FDU-2100, Japan). The crystals were stored at −20°C until analysis. The stock solution formulated 1 g of crystals in 1 ml PBS.

### 2.2. RGM 1 Cells Cultured in Acidic Medium

The RGM 1 cell line (RCB0876) was obtained from Prof. Hirofumi Matsui (University of Tsukuba, Japan). RGM 1 cells were cultured in DMEM/F12 medium (pH 7.4) at 37°C in 5% humidity in a CO_2_ incubator, and the medium was changed every two days. First, RGM 1 cells were seeded in 96-well plates at a density of 2 × 10^4^ cells/well and pretreated with *Magnolia officinalis* water extract (0, 0.1, 1, 2, 5, or 10 mg/ml) or sucralfate (3 mg/ml) as a positive control for 2 h. The medium was changed to DMEM/F12 medium (pH 4.0) for 40 min to simulate acid damage.

Additionally, 2 × 10^4^ cells/well were seeded in 96-well plates and pretreated with *Magnolia officinalis* water extract (0, 0.1, 1, 2, 5, or 10 mg/ml) or sucralfate (3 mg/ml) for 2 h in DMEM/F12 medium (pH 7.4). Then, the cytotoxicity of *Magnolia officinalis* water extract or sucralfate was evaluated with MTT assays.

### 2.3. Protection from *Magnoliae officinalis* Water Extract in RGM 1 Cells Cultured in Acidic Medium

After 40 min of acidic medium stimulation, the cells were incubated for 2 h with 0.5 mg/ml of MTT and dissolved in serum-free medium. Then, DMSO (100 *μ*l) was added with gentle shaking for 10 min so that complete dissolution was achieved. Aliquots of the resulting solutions were transferred to 96-well plates, and absorbance was recorded at 595 nm using the microplate spectrophotometer system (Spectra max190-Molecular Devices). The results were analyzed with the Soft max pro software (version 2.2.1) and are presented as percentages of the control values.

### 2.4. ROS Production of RGM 1 Cells Cultured in Acidic Medium

We used 0.1, 1, and 2 mg/ml of *Magnolia officinalis* water extract to perform the experiment. RGM 1 cells were seeded in 24-well plates at a density of 2 × 10^5^ cells/well and pretreated with *Magnolia officinalis* water extract (0, 0.1, 1, or 2 mg/ml) for 2 h. Then, the medium was changed to DMEM/F12 medium (pH 4.0) for 10 min. We then washed with PBS and added 10 *μ*M 2,7-dichlorofluorescein diacetate (H2DCF-DA, Invitrogen, USA) dissolved in PBS. Then, the cells were incubated at 37°C for 30 min. After removing the solution, the cells were incubated in MEM medium (without phenol red) for 5 min and the cells were collected. The positive cell counts and mean fluorescence intensities were analyzed by flow cytometry (Beckman Coulter Epics XL-MCL, USA) using 488 nm excitation and 526 nm receiving fluorescence signals.

### 2.5. Animal Model of Gastric Ulcer

This study was approved by the Animal Care and Use Committee of National Pingtung University of Science and Technology (approval number: IACUC-NPUST-106-053). Shay's rat ulcer model was adopted and modified [8]. Normal six-week-old ICR mice were purchased from BioLASCO Taiwan Co., Ltd. (Taipei, Taiwan) and housed under constant temperature, humidity, and illumination (12 hours of light and dark). Water and standard diet were made available ad libitum. After an adaptation period, the mice were divided into four groups (*n* = 10, each group). One group was a sham group, and the other groups were administrated daily with saline, *Magnolia officinalis* water extract (100 mg/kg), or ranitidine (100 mg/kg) via oral gavage for 28 days.

The mice were fasted on day 28. After the pretreatment period of 1 h on the last day, the mice were anesthetized with Zoletil (50 mg/kg) (Virbac, New Zealand). The abdomen was opened by a small midline incision below the xiphoid process, and the pylorus portion of the stomach was slightly lifted out and ligated by surgical sutures. Precaution was taken to avoid traction to the pylorus or damage to its blood supply. The stomach was placed carefully in the abdomen and the wound was sutured by interrupted sutures. Then, 4 h after pylorus ligation, the mice were euthanized by cervical dislocation after anesthetization, and the stomach was removed. The volume, pH, and total acidity of gastric fluid were determined. The stomach was then incised along the greater curvature and observed to elucidate the ulcer index. The ulcer index was counted using a magnifying glass, and the diameter of the ulcers was measured using a vernier caliper.

### 2.6. Ulcer Index Determination

The ulcer index was modified by following the scoring method of Suzuki et al. [9] and judged by Prof. Ching-Dong Chang (pathologist, National Pingtung University of Science and Technology). The formula was as follows:

Protection ration (%) = (Control ulcer index – Test ulcer index)/Control ulcer index.

### 2.7. Biochemical Estimation

The CB2 gene, TNF-*α*, and IL-6 concentration of stomach tissue were analyzed by RT-PCR and ELISA kits (R&D Systems, USA). The primer sequences of CB2 were as follows: forward 5′-TGATCCTGAGCAGTGGCCAGCAGA, reverse 5′-AGGTCATGGTCACACTGCCGATCT. The primer sequences of *β*-actin were as follows: forward 5′-CTGGTCGTACCACAGGCATT, reverse 5′-CTTTGATGTCACGCACGATTT.

### 2.8. Statistical Analysis

All data were analyzed to determine the statistical significance of differences among groups. Student's *t*-test, Mann–Whitney *U* test, and one-way analysis of variance with Bonferroni's post hoc test were used to assess the differences between groups. *P* < 0.05 was considered statistically significant. The Statistical Package for Social Sciences (SPSS, version 19.0; Chicago, IL, USA) was used to perform all statistical analyses.

## 3. Results

### 3.1. Cell Viability of RGM 1 Cells in Acidic Medium

The *Magnolia officinalis* water extract (0.1, 1, 2, 5, or 10 mg/ml) and sucralfate (3 mg/ml) did not possess cytotoxicity in RGM 1 cells cultured in normal medium (data not shown).

**Figure 2:**
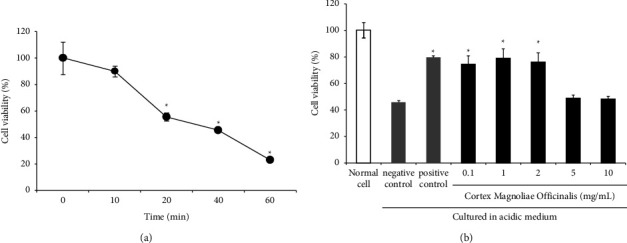
Cell viability of RGM 1 cells. (a) The time course of RGM 1 cell incubation in the acidic medium to induce cell damage. Cells were cultured in normal medium (pH 7.4) for 2 h and acidic medium (pH 4.0). (b) RGM 1 cells were pretreated with 100 *μ*L of various concentrations (0.1, 1, 2, 5, and 10 mg/mL) of *Magnolia officinalis* water extract or sucralfate (3 mg/mL) as the positive control and cultured in normal medium for 2 h and acidic medium for 40 min. Data are presented as a relative percentage versus the mean of the control group (100%) and the mean ± S.E. from three independent experiments. ^∗^*p* < 0.05 vs. negative control.

When RGM 1 cells were cultured in acidic medium for 10 min, the cell viability was significantly decreased. After pretreatment with sucralfate (3 mg/ml) or *Magnolia officinalis* water extract (0.1, 1, or 2 mg/ml), the cell viability of RGM 1 cells was increased in the acidic medium stimulation, while 5 and 10 mg/ml of *Magnolia officinalis* water extract did not increase the cell viability (Figure 2). Therefore, we suggest that *Magnolia officinalis* water extract in low doses, as well as sucralfate, which possessed protective effects for gastric mucosa epithelial cells damaged by gastric acid.

### 3.2. Antioxidant Effect of *Magnolia officinalis* Water Extract *In Vitro*

The reactive oxygen species (ROS) production of RGM 1 cells induced by acidic medium for 10 min was more significant than that for 40 min (Table 1). We found that 0.1 mg/ml of *Magnolia officinalis* water extract and 3 mg/ml of sucralfate effectively decreased the ROS production (Table 2). However, *Magnolia officinalis* water extract did not exhibit dose-dependent efficacy in this test.

### 3.3. Ulcer Index Determination

**Figure 3:**
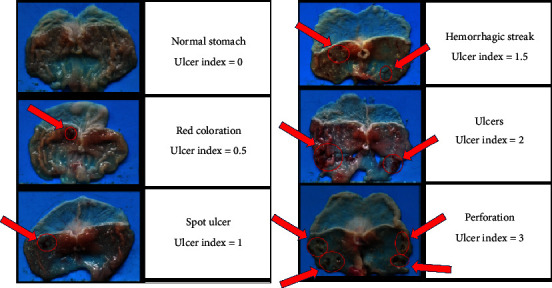
Morphological appearances and ulcer index of different ulcer index on gastric mucosa.

Figure 3 shows the modified scoring method of the ulcer index, which could more easily assesss the severity of gastric ulcers. After pylorus ligation for 4 h, the gastric ulcers of the saline group were induced significantly, compared with those of the sham group. However, in the ranitidine or *Magnolia officinalis* water extract group, the gastric ulcers were reduced significantly. Therefore, *Magnolia officinalis* water extract as well as ranitidine, ameliorated gastric mucosa damage from gastric acid (Table 3).

### 3.4. Biochemical Parameters *In Vivo*

After pylorus ligation for 4 h, the gastric juice secretion and pH value of gastric juice in the saline group were higher than those of the sham group (Table 3). Therefore, the pylorus ligation was successful for inducing gastric ulcers. In contrast, in the ranitidine or *Magnolia officinalis* water extract groups, those parameters were significantly ameliorated (Table 3). CB2 activation is known to reduce inflammation. Although the CB2 gene expression was not changed by pylorus ligation between the saline and sham groups, ranitidine or *Magnolia officinalis* water extract could enhance CB2 gene expression (Table 4). Meanwhile, after pylorus ligation, the concentrations of the inflammatory cytokines, TNF-*α* and IL-6 were increased. Ranitidine or *Magnolia officinalis* water extract reduced the levels of those cytokines significantly (Table 4). Therefore, ranitidine or *Magnolia officinalis* water extract might regulate the inflammation through stimulating CB2 gene (Table 4).

## 4. Discussion

This study demonstrated that *Magnolia officinalis* water extract could prevent the RGM 1 gastric mucosa epithelial cell line from being damaged by gastric acid. It could also ameliorate gastric acid-induced gastric ulcers and inflammation in mice. These findings prove the therapeutic benefits of *Magnolia officinalis* water extract in treating gastric ulcers.

*Magnolia officinalis* is applied widely in Asian traditional medicine for its multiple uses, including sedative, antispastic, antibiotic, antioxidant, and anti-inflammatory effects, as well as for its ability to treat tract ulcer diseases [10, 11]. The major substances of *Magnolia officinalis*, magnolol and honokiol, are responsible for its beneficial properties. Many food safety authorities have evaluated *Magnolia officinalis* and its extract and indicated their safety. Furthermore, Sarrica et al. [11] demonstrated that using *Magnolia officinalis* and its extract for more than one year did not result in considerable adverse effects. Additionally, our result demonstrated that *Magnolia officinalis* water extract (0.1–10 mg/ml) had no cytotoxicity in the RGM 1 cell line, which was consistent with the findings of Sarrica et al.

Under the stimulation of excessive gastric acid secretion for a prolonged period, the gastric mucosa will experience serious damage, eventually resulting in gastric ulcers and perforation [12]. Protecting the gastric mucosa is beneficial for treating ulcers. Sucralfate, a mucosal protectant, is an aluminum salt of sucrose octasulfate that can be activated with gastric acid to form a viscous material that acts as an acid buffer. This protectant attaches to proteins on the surface of the ulcers to form a stable complex to prevent further gastrointestinal ulcers from gastric acid, pepsin, or bile acid [13]. Sucralfate can also stimulate the production of prostaglandins and gastric mucus to protect the tract [14]. In this study, *Magnolia officinalis* water extract, as well as sucralfate, could protect the RGM 1 cell line from damage induced by an acidic medium. Therefore, we suggest that *Magnolia officinalis* water extract could be used as a mucosal protectant like sucralfate.

The major risk factors of peptic ulcers, such as stress, smoking, alcohol, and drugs are related to tissue damage caused by ROS production [15]. When RGM 1 cells were cultured in acidic medium, ROS production was observed. However, after treating these cells with *Magnolia officinalis* water extract, ROS generation was inhibited. We also confirmed that sucralfate could decrease ROS production, which was consistent with the findings of Sato et al. [16]. We believe that one of the mucosal protective mechanisms of *Magnolia officinalis* water extract is related to inhibiting ROS generation.

In this study, we performed pylorus ligation surgery to induce gastric ulcers and observed the results of Shays' method. After pretreatment with *Magnolia officinalis* water extract or ranitidine, the ulcer index was decreased significantly, indicating that both of these treatments could inhibit gastric acid-induced gastric ulcers. Furthermore, in the analysis of the tissue biochemical parameters, the CB2 gene expression was not changed significantly, but TNF-*α* and IL-6 concentrations were increased by pylorus ligation surgery. Cannabinoid receptor (CB) can be roughly divided into two types: CB1 and CB2. CB1 receptor is predominantly expressed in the central nervous system (CNS) to mediate the psychotropic and behavioral effects of cannabinoid. In comparison, CB2 receptor is widely distributed in several tissues, such as CNS, cardiovascular, respiratory, hepatic, and reproductive systems to affect their function and immunity. In the gastrointestinal tract, CB2 modulates visceral motility, sensitivity, and inflammation. The downregulation of CB2 is proved to enhance gastric inflammation and ulcers [17]. Some reports have demonstrated that *Magnolia officinalis* can activate CB2 [18, 19]. In this study, we were the first to prove that *Magnolia officinalis* water extract and ranitidine could stimulate CB2 gene expression and inhibit inflammatory cytokines in a mouse model of gastric ulcers. We believe this to be direct evidence of the mechanism of *Magnolia officinalis* in treating gastrointestinal ulcers.

## 5. Conclusion

**Figure 4:**
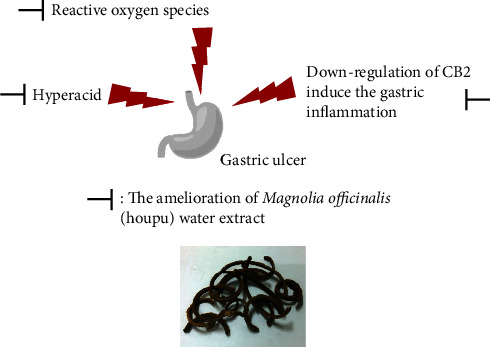
The hypothesis mechanisms of *Magnolia officinalis* water extract ameliorating gastric ulcer. *Magnolia officinalis* (houpu) water extract not only enhanced the pH value of stomach acid but also ameliorated the ulcer index and inflammation and increased CB2 expression effectively. These results suggest that *Magnolia officinalis* water extract might be used to decrease the incidence of gastric ulcer.

*Magnolia officinalis* water extract exhibited gastric mucosal protection, inhibited ROS production, prevented ulcer formation, and reduced inflammation in gastric ulcers (Figure 4). We developed the water extraction of *Magnolia officinalis* and proved its efficiency and mechanisms in preventing gastric ulcers. We believe our findings will contribute to a wider application of *Magnolia officinalis* in traditional medicine.

## Figures and Tables

**Table 1:** Time course of ROS production of RGM 1 cells cultured in acidic medium.

Time (min)	ROS production (%)
0	28.5 ± 5.1
10	53.6 ± 3.8
20	47.3 ± 3.6
40	9.4 ± 2.3

Cells (2 × 10^5^ cells/well) incubated in acidic medium (pH 4.0) for 0, 10, 20, and 40 min. ROS levels were estimated by comparing the DCF fluorescence intensity using flow cytometry.

**Table 2:** Effect of *Magnolia officinalis* water extract on ROS production of RGM 1 cells.

*Magnolia officinalis* water extract (mg/ml)	ROS production (%)
0	53.6 ± 3.8
0.1	38.2 ± 4.5^∗^
1	49.2 ± 4.3
2	48.4 ± 3.5
5	50.3 ± 4.1
10	47.6 ± 4.5
Sucralfate 3 mg/ml	33.7 ± 3.6^∗^

Cells were pretreated with *Magnolia officinalis* water extract or sucralfate in normal medium (pH 7.4) for 2 h and then exposed to the acidic medium (pH 4.0) for 10 minutes. Values represent as the mean ± *S*.E. (*n* = 3). 0 mg/ml *Magnolia officinalis* water extract, as a negative control. ^∗^*p* < 0.05 vs. negative control.

**Table 3:** Protective effects of *Magnolia officinalis* water extract or ranitidine in pylorus ligation ICR mice.

	Evaluation index			
Groups	Ulcer index	Protection ratio (%)	Gastric juice secretion (ml)	pH value of gastric juice
Sham	0	—	0.14 ± 0.03	4.94 ± 0.52
Saline	0.75 ± 0.63^#^	0	0.31 ± 0.02^#^	3.12 ± 0.55^#^
*Magnolia officinalis* water extract	0.17 ± 0.35^∗^	77.7^∗^	0.19 ± 0.07^∗^	4.21 ± 0.47^∗^
Ranitidine	0.25 ± 0.35^∗^	66.6^∗^	0.23 ± 0.04^∗^	4.92 ± 0.74^∗^

Mice were pretreated with *Magnolia officinalis* water extract or ranitidine (100 mg/kg). The control group received an equal volume of saline for 28 days, and then pylorus ligation was performed. After 4 h, the mice were euthanized. Values represent the mean ± S.E. (*n* = 10). ^#^*p* < 0.05 vs. sham group. ^∗^*p* < 0.05 vs. saline group.

**Table 4:** Biochemical parameters of pylorus ligation in ICR mice.

	Biochemical parameters		
Groups	CB2 gene expression (%)	TNF-*α* (pg/ml)	IL-6 (pg/ml)
Sham	100	9.1 ± 3.4	12.8 ± 4.1
Saline	113.4 ± 22.8	21.1 ± 2.6^#^	245.8 ± 32.6^#^
*Magnolia officinalis* water extract	271.3 ± 29.1^∗^	17.5 ± 2.1^∗^	157.2 ± 20.3^∗^
Ranitidine	219.7 ± 26.3^∗^	16.2 ± 2.9^∗^	208.2 ± 27.5^∗^

Mice were pretreated with *Magnolia officinalis* water extract or ranitidine (100 mg/kg). The control group received an equal volume of saline for 28 days, and then pylorus ligation was performed. After 4 h, the mice were euthanized. Values represent the mean ± *S*.E. (*n* = 10). ^#^*p* < 0.05 vs. sham group. ^∗^*p* < 0.05 vs. saline group.

## Data Availability

The data used to support the findings of this study are available from the corresponding author upon reasonable request.
